# Small airway dysfunction in pneumoconiosis: a cross-sectional study

**DOI:** 10.1186/s12890-022-01929-9

**Published:** 2022-04-28

**Authors:** Yali Fan, Ruimin Ma, Xuqin Du, Dandan Chai, Shuangli Yang, Qiao Ye

**Affiliations:** Department of Occupational Medicine and Toxicology, Clinical Center for Interstitial Lung Diseases, Beijing Institute of Respiratory Medicine, Beijing Chao-Yang Hospital, Capital Medical University, Beijing, 100020 China

**Keywords:** Small airway dysfunction, Pneumoconiosis, Dust exposure, Prevalence, Risk factor

## Abstract

**Background:**

Although several histological studies have documented airway inflammation and remodelling in the small airways of dust-exposed workers, little is known regarding the prevalence and risk factors of small airway dysfunction (SAD) in pneumoconiosis. The present study investigated the prevalence and characteristics of spirometry-defined SAD in pneumoconiosis and assessed the risk factors for associated with SAD.

**Methods:**

A total of 1255 patients with pneumoconiosis were invited to participate, of whom 1115 patients were eligible for final analysis. Spirometry was performed to assess SAD using the following three indicators: maximal mid-expiratory flow and forced expiratory flow 50% and 75%. SAD was defined as at least two of these three indicators being less than 65% of predicted value. Logistic regression analyses were used to analyse the relationships between clinical variables and SAD.

**Results:**

Overall, 66.3% of patients with pneumoconiosis had SAD, among never-smokers the prevalence of SAD was 66.7%. The proportion of SAD did not differ among the subtypes of pneumoconiosis. In addition, SAD was present across the patients with all stages of pneumoconiosis. Even among those with forced expiratory volume in 1 s (FEV_1_) ≥ 80% and FEV_1_/forced vital capacity ratio ≥ 70%, 40.8% of patients had SAD. Patients with SAD were older than patients without SAD, more likely to be women and heavy smokers. Importantly, patients with SAD had more severe airflow obstruction, air trapping, and diffusion dysfunction. All patients with both pneumoconiosis and chronic obstructive pulmonary disease had SAD. Based on multivariate analysis, overall, aged 40 years and older, female sex, heavy smoking, body mass index ≥ 25.0 kg/m^2^ and pneumoconiosis stage III were significantly associated with increased risk of SAD. Among the never smokers, risk factors for SAD included female sex, BMI ≥ 25.0 kg/m^2^, pneumoconiosis stage II and stage III

**Conclusion:**

Spirometry-defined SAD is one of the common functional abnormalities caused by occupational dust exposure and should be taken into account when monitoring respiratory health of workers to guide the early precautions and management in pneumoconiosis.

**Supplementary Information:**

The online version contains supplementary material available at 10.1186/s12890-022-01929-9.

## Background

Pneumoconiosis is an irreversible, potentially fatal dust-related lung disease caused by inhalation of mineral dust [1–3]. This disease remains one of the major occupational health concerns, especially in developing countries and territories [1, 3–5]. In 2019, more than 880,000 patients with pneumoconiosis were reported in China, accounting for 88.9% of the total number of occupational diseases [6]. In 2016, 21,488 deaths were estimated to be due to pneumoconiosis on a global scale [7]. Occupational dust exposure induces inflammation and fibrosis in the lungs, which can affect the entire respiratory tract, including the large and small airways [2].

The small airways are considered the main site of airflow limitation in obstructive lung disease [8]. A recent large epidemiological study showed that the prevalence of small airway dysfunction (SAD) (defined as the presence of at least two of these three indicators less than 65% of predicted values: maximal mid-expiratory flow (MMEF), forced expiratory flow (FEF) 50%, and FEF 75%) was more than 40% among Chinese adults aged 20 years or older, accounting for more than 400 million people in China [9]. Histological data suggested that collagen and hyaluronan were increased in the small airway in chronic obstructive pulmonary disease (COPD), and structural abnormalities in the small airways may precede pathological evidence of emphysematous destruction [10, 11]. SAD is a common early feature of COPD, all patients with COPD had SAD [9–11].

Long-term exposure to mineral dust leads to the development of obstructive lung diseases, such as COPD [2, 12, 13]. Our previous studies showed that COPD was highly prevalent in patients with pneumoconiosis, especially in silicosis and coal workers’ pneumoconiosis [14]. Notably, both physiological and structural abnormalities in small airways among workers exposed to a variety of mineral dusts have been observed [13, 15, 16]. Our published data showed that the predicted percentages of FEF 25%, FEF 50% and FEF 75% were all significantly lower in asbestosis, suggesting that SAD was present [17]. Previous research on SAD has primarily focused on the general population or patients with COPD or asthma. However, the proportion and risk factors for SAD in pneumoconiosis have not been determined. The present study aimed to estimate the prevalence of pre-bronchodilator SAD and assessed risk factors for its in pneumoconiosis using a spirometric definition of the disease.

## Methods

### Study design and participants

This research was a cross-sectional study and designed according to the Strengthening the Reporting of Observational Studies in Epidemiology (STROBE) statement [18]. From January 2007 to November 2020, 1255 newly diagnosed pneumoconiosis patients were recruited from Beijing Chaoyang Hospital. A multidisciplinary diagnostic review was performed to confirm the diagnosis of pneumoconiosis according to the criteria for pneumoconiosis of the International Labour Organization (ILO) classification [19]. The multidisciplinary diagnostic panel included at least pulmonologist, pathologists, and radiologist. Diagnosis of pneumoconiosis was based on relevant occupational exposure history and clinic-radiological correlations. Patients with pulmonary malignant tumours, acute pulmonary infection, pulmonary tuberculosis, asthma, bronchiectasis, pneumothorax or those without spirometry available for physical review were excluded.

This study was conducted in accordance with the ethical standards of Beijing Chaoyang Hospital and World Medical Association Declaration of Helsinki and was approved by the Institutional Review Board (IRB) of Beijing Chaoyang Hospital. Informed consent was documented in writing.

### Data collection

The following data were collected: demographics, medical history, smoking (including smoking status, cigarettes smoked per day, and pack-years smoked), family history, and detailed occupational history (including type of exposure and the start and end dates of employment). Smoking status was self-reported and classified as current smoker (current smoking or cessation < 12 months), former smoker (cessation ≥ 12 months previously) and never-smoker. Smoking intensity was analysed as both a categorical (0 pack-years, 1–19 pack-years and ≥ 20 pack-years) and continuous variable. Heavy smoking was defined as having smoked 20 or more pack-years. Both categorical (< 18.5 kg/m^2^, 18.5–24.9 kg/m^2^, and ≥ 25.0 kg/m^2^) and continuous variables to analyse body mass index (BMI) were also used for analysis.

### Pulmonary function tests

The patients with pneumoconiosis underwent pulmonary function tests. All the pulmonary function tests data based on criteria from the American Thoracic Society and European Respiratory Society criteria were reviewed centrally an expert panel [20]. Unreliable spirometric data were excluded. Trained technicians performed pulmonary function examinations using spirometry, whole body plethysmography, and single-breath diffusing capacity for carbon monoxide. SAD in patients with pneumoconiosis was assessed on the basis of three indicators of lung function, namely, pre-bronchodilator MMEF, FEF at 50% of vital capacity, and FEF at 75% of vital capacity according to the recommendations of a previous study [9]. SAD was defined as present if at least two of these three indicators were less than 65% of predicted values. Based on previous studies, the cutoff value of 65% predicted value was chosen, especially in the Chinese population [9]. COPD was diagnosed according to medical history and forced expiratory volume in 1 s (FEV_1_)/forced vital capacity (FVC) ratio < 70% after bronchodilation [21]. A bronchial challenge test was performed in patients with FEV_1_ above 60%. Airway hyperresponsiveness was tested using a bronchial challenge test when a provocative methacholine concentration (4 mg/mL or less) causing a 20% decrease in FEV_1_ was considered positive.

### Stages of pneumoconiosis

Each patient’s chest radiographs were independently reviewed by two thoracic radiologists who were blinded to the clinical information with good interobserver correlation (0.81). All disagreements were resolved through consensus. Pneumoconiosis was classified into three stages according to the density and distribution of small and large opacities on the posterior chest radiograph, using a national criterion on the diagnosis of occupational pneumoconiosis (GBZ 70-2015) [22], which is in line with ILO classification guideline (Additional file 1: Methods) [19].

### Sample size calculation

Based on a previous study, this study assumed that the prevalence of SAD in pneumoconiosis was 43.5% [9]. Using the formula, the sample size was 499 [9]. If fixed precision (*d*) was specified, using the formula, the sample size was 385. Furthermore, this study demonstrated the prevalence of SAD in the never-smokers subgroup. Thus, the final sample sizes were 895–1160 according to the proportion of never-smokers in patients with pneumoconiosis from Beijing Chao-Yang Hospital. We calculated sample sizes with PASS software (NCSS, Kaysville, UT, USA).

### Statistical analysis

SPSS Statistics V.23 (IBM Inc., Chicago, Illinois, USA) and GraphPad Prism V8 (GraphPad Software, La Jolla, USA) were used to perform the statistical analyses and to make plots. Data are expressed as the median (interquartile range) or number and percentage. The Mann–Whitney U test was conducted to determine the differences in nonnormally distributed continuous variables. The chi-square test or Fisher’s exact test was used for categorical variables, when appropriate. Spearman's (nonparametric) correlation was used to assess the relationship between pulmonary function variables. Logistic regression analyses with odds ratios (ORs) with 95% confidence intervals (CIs) were applied to investigate potential risk factors for SAD in all pneumoconiosis patients and in never-smokers. Multivariable model was adjusted for age, sex, smoking exposure, BMI, the duration of exposure, exposure type and stage of pneumoconiosis. A *p* value of less than 0.05 was considered to be significant.

## Results

### Demographics

**Fig. 1:**
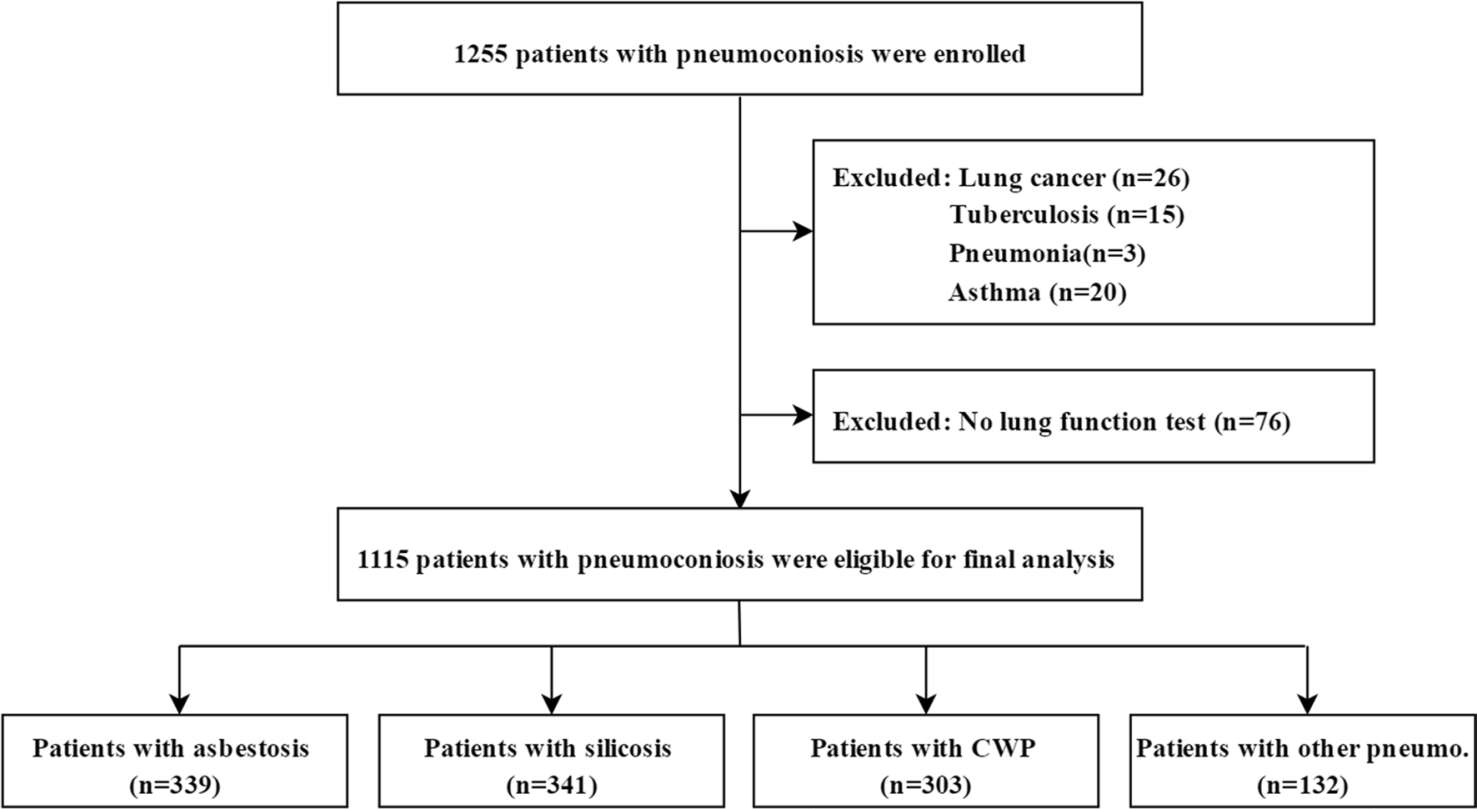
Flow chart of the enrolled patients. *CWP* coal workers’ pneumoconiosis

Of 1255 patients with pneumoconiosis who were initially recruited, 1115 patients with complete data were eligible for inclusion in the final analysis (Fig. 1). Among these patients, 339 patients with asbestosis, 341 with silicosis, 303 with coal workers’ pneumoconiosis and 132 with other pneumoconiosis were enrolled. The median age was 58 (IQR 50–67) years, the majority of patients were men (74.3%, 829/1115), and 620 (55.6%) patients had a history of smoking. The characteristics of the patients with pneumoconiosis are presented in Table 1.

**Table 1:** Demographic characteristics of 1115 pneumoconiosis patients seen in the Beijing Chaoyang Hospital by type of pneumoconiosis

	All	Asbestosis	Silicosis	Coal workers’ pneumoconiosis	Other pneumoconiosis	*p* Value
n	1115	339	341	303	132	
Age, yrs	58.0 (50.0–67.0)	67.0 (62.0–73.0)	55.0 (48.0–65.0)	53.0 (49.0–58.0)	48.5 (44.0–59.8)	< 0.001
*Sex*						< 0.001
Men	829 (74.3)	153 (45.1)	253 (74.2)	299 (98.7)	124 (93.9)	
Women	286 (25.7)	186 (54.9)	88 (25.8)	4 (1.3)	8 (6.1)	
BMI, kg/m^2^	25.1 (22.9–27.6)	26.1 (24.1–28.9)	24.5 (22.5–26.9)	24.6 (21.8–26.9)	24.6 (22.5–26.8)	< 0.001
*Smoking exposure, pack-yrs*						< 0.001
0	495 (44.4)	221 (65.2)	150 (44.0)	84 (27.7)	40 (30.3)	
1–19	372 (33.4)	65 (19.2)	97 (28.4)	143 (47.2)	67 (50.8)	
≥ 20	248 (22.2)	53 (15.6)	94 (27.6)	76 (25.1)	25 (18.9)	
Cumulative pack-yrs	15.0 (5.0–25.0)	15.0 (5.0–30.0)	18.4 (7.5–30.0)	12.5 (4.8–22.5)	10.0 (3.2–20.0)	< 0.001
Duration of exposure, yrs	12.0 (6.0–21.0)	9.0 (5.0–23.0)	13.9 (7.0–24.0)	15.0 (7.0–20.0)	12.0 (8.0–18.8)	0.025
*Stage of pneumo*						< 0.001
I	599 (53.7)	231 (68.1)	156 (45.7)	113 (37.3)	99 (75.0)	
II	286 (25.7)	91 (26.8)	82 (24.0)	88 (29.0)	25 (18.9)	
III	230 (20.6)	17 (5.0)	103 (30.2)	102 (33.7)	8 (6.1)	

Data was presented as median (IQR) or n (%)

*BMI* body-mass index, *IQR* interquartile range

### Prevalence of SAD in pneumoconiosis

Overall, 66.3% (739/1115) of the study population had SAD. The prevalence of SAD did not significantly differ among the various subtypes (Table 2). Women had a higher prevalence than men (73.4% vs. 63.8%, *p* = 0.003), and the difference was statistically significant. The prevalence of SAD increased with age and smoking pack-years. However, the prevalence of SAD did not show a significant difference between never-smokers and smokers (66.7% vs. 66.0%, *p* = 0.806). In addition, SAD was present across the patients with all stages of pneumoconiosis and was 61.1% in stage I, 66.4% in stage II and 79.6% in stage III (*p* < 0.001). Interestingly, all patients with both pneumoconiosis and COPD had SAD.

**Table 2:** Distribution of the general characteristics in the study sample and prevalence rates of small airway dysfunction by the general characteristics

	All		Prevalence of SAD		
	n	%	n	%	*p* Value
Overall	1115	100	739	66.3	
*Pneumoconiosis*					0.059
Asbestosis	339	30.4	232	68.4	
Silicosis	341	30.6	239	70.1	
Coal workers’ pneumoconiosis	303	27.2	189	62.4	
Other pneumoconiosis	132	11.8	79	59.8	
*Age, yrs*					< 0.001
20–39*	40	3.6	17	42.5	
40–49	223	20.0	123	55.2	
50–59	334	30.0	230	68.9	
60–69	315	28.3	215	68.3	
≥ 70	203	18.2	154	75.9	
*Sex*					0.003
Men	829	74.3	529	63.8	
Women	286	25.7	210	73.4	
Smoking history					0.559
Never-smoker	495	44.4	330	66.7	
Former smoker	312	28.0	212	67.9	
Current smoker	308	27.6	197	64.0	
*Smoking exposure, pack-yrs*					0.017
0	495	44.4	330	66.7	
1–19	372	33.4	229	61.6	
≥ 20	248	22.2	180	72.6	
*BMI*^*‡*^*, kg/m*^*2*^					0.114
< 18.5	16	1.4	12	75.0	
18.5–24.9	531	47.6	336	63.3	
≥ 25.0	568	50.9	391	68.8	
*Duration of exposure, yrs*					0.949
~ 4	168	15.1	112	66.7	
5–10	331	29.7	219	66.2	
11–15	166	14.9	113	68.1	
16 ~	450	40.4	295	65.6	
*Stage of pneumoconiosis*					< 0.001
I	599	53.7	366	61.1	
II	286	25.7	190	66.4	
III	230	20.6	183	79.6	
COPD	345	30.9	345	100	< 0.001

*SAD* small airway dysfunction, *BMI* body-mass index, *COPD* chronic obstructive pulmonary disease

*Age groups 20–29 years and 30–39 years were combined because of small numbers of patients

^‡^The patients with BMI < 18.5 kg/m^2^ means underweight, 18.5–24.9 kg/m^2^ means normal range, and ≥ 25.0 kg/m^2^ means overweight and obese

After restricting analyses to never-smokers, SAD was present in 66.7% (330/495) of patients with pneumoconiosis, and the prevalence of SAD according to age, sex, smoking pack-years, and stage of pneumoconiosis was not substantially altered (Additional file 1: Table S1).

**Fig. 2:**
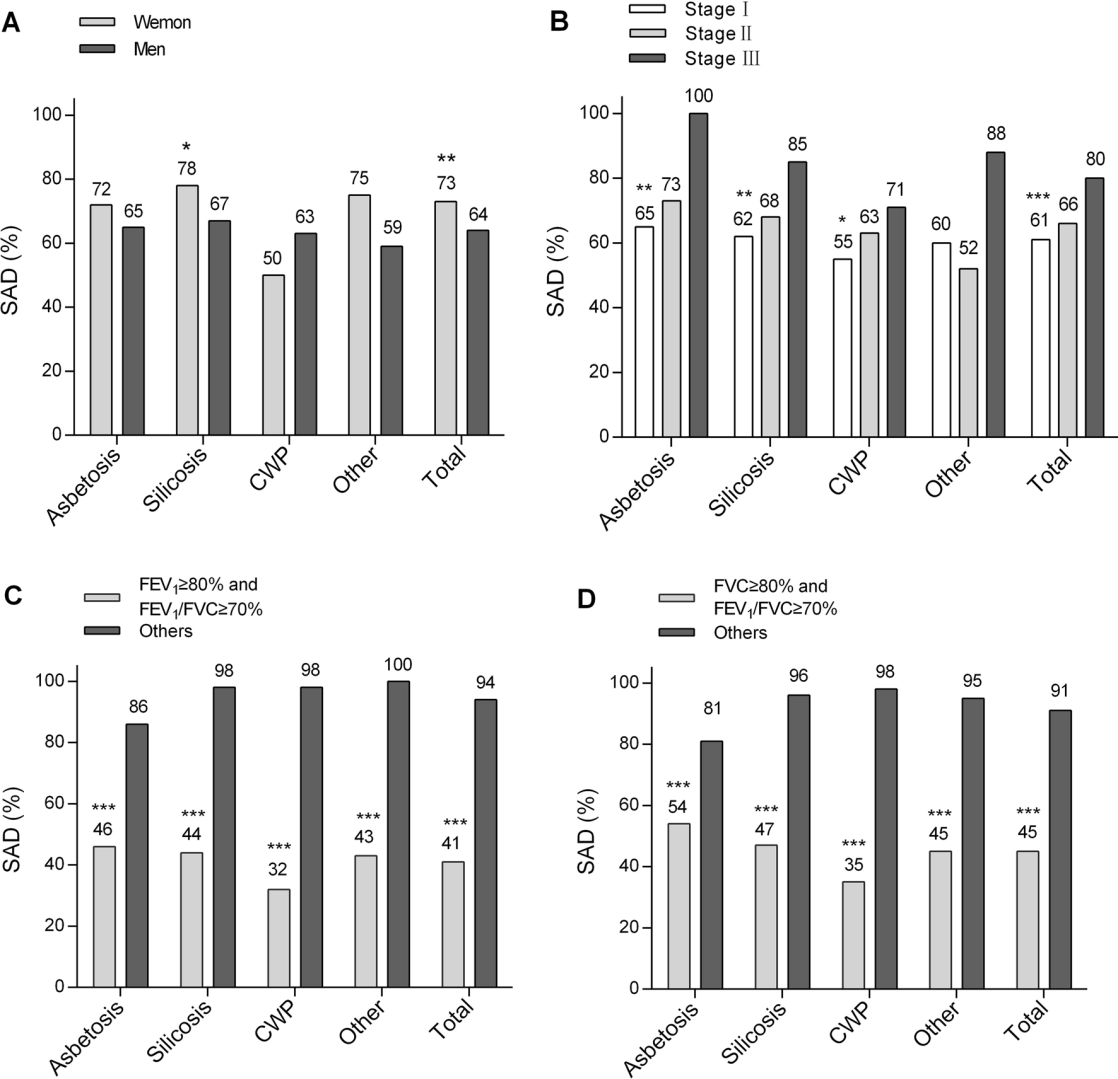
The prevalence of small airway dysfunction in various subtypes of pneumoconiosis and total patients. **A** *p* values for women versus men, **B** *p* values for comparison across stages of pneumoconiosis, **C** *p* values for FEV_1_ ≥ 80% and FEV_1_/FVC ≥ 70% group versus others, **D** *p* values for FVC ≥ 80% and FEV_1_/FVC ≥ 70% group versus others. *SAD* small airway dysfunction; *, *p* < 0.05; **, *p* < 0.01; ***, *p* < 0.001

In the analysis of subtypes of pneumoconiosis, the prevalence of SAD is presented in Fig. 2. The prevalence of SAD increased steadily with stages of pneumoconiosis in asbestosis, silicosis, and coal workers’ pneumoconiosis.

In addition, 237 patients had SAD when the patients had FEV_1_ ≥ 80% and FEV_1_/FVC ratio ≥ 70% (21.3% of all participants, and 32.1% of all those with SAD) (Additional file 1: Table S2). Specifically, among 581 patients with FEV_1_ ≥ 80% and FEV_1_/FVC ratio ≥ 70%, 40.8% of patients with pneumoconiosis had SAD. Similarly, among 598 patients with FVC ≥ 80% and FEV_1_/FVC ratio ≥ 70%, 44.8% of patients had SAD (Additional file 1: Table S3).

### Characteristics of the patients with small airway dysfunction

Patients with SAD were older (median 59.5 years old) than patients without SAD and were more likely to be women (Table 3), and the differences were statistically significant. Compared with patients without SAD, those with SAD had a significantly higher number of cigarette pack-years and a significantly higher proportion of heavy smokers, but the proportion of never-smokers was not significantly different between those with and without SAD (44.7% vs. 43.9%, *p* = 0.806). Patients with SAD also had more frequent cough and expectoration than patients without SAD, and the differences were statistically significant.

**Table 3:** General characteristics of the study sample stratified in relation to the presence or absence of small airway dysfunction

	Small airway dysfunction		*p* Value
	Presence (n = 739)	Absence (n = 376)	
Age, yrs	59.5 (51.0–68.0)	55.0 (47.0–65.8)	< 0.001
*Sex*			0.003
Men	529 (71.6)	300 (79.8)	
Women	210 (28.4)	76 (20.2)	
*Smoking exposure, pack-yrs*			0.017
0	330 (44.7)	165 (43.9)	
1–19	229 (31.0)	143 (38.0)	
≥ 20	180 (24.4)	68 (18.1)	
Cumulative pack-yrs	15.0 (5.0–28.1)	10.0 (5.0–20.0)	< 0.001
BMI, kg/m^2^	25.3 (22.9–27.7)	24.8 (22.9–27.5)	0.439
Duration of exposure, yrs	12.0 (6.0–21.0)	12.0 (6.0–22.0)	0.840
*Stage of pneumoconiosis*			< 0.001
I	366 (49.5)	233 (62.0)	
II	190 (25.7)	96 (25.5)	
III	183 (24.8)	47 (12.5)	
*Types of pneumoconiosis*			0.059
Asbestosis	232 (31.4)	107 (28.5)	
Silicosis	239 (32.3)	102 (27.1)	
Coal workers’ pneumoconiosis	189 (25.6)	114 (30.3)	
Other pneumoconiosis	79 (10.7)	53 (14.1)	
Cough	595 (80.5)	267 (71.0)	< 0.001
Expectoration	373 (50.5)	154 (41.0)	0.003
Dyspnea	453 (61.3)	239 (63.6)	0.461

Data was presented as n (%) or median (IQR)

*SAD* small airway dysfunction, *BMI* body-mass index

Patients with SAD had more airflow limitation, air trapping, and diffusion dysfunction (Additional file 1: Table S4). Notably, among 739 patients with SAD, 408 (55.2%) had FEV_1_ less than 80%, 345 (46.7%) patients had FEV_1_/FVC ratio less than 70%, 251 (34.0%) had both FEV_1_ less than 80% and FEV_1_/FVC ratio less than 70%. However, only 8.2% (31/376) of the patients without SAD had FEV_1_ less than 80%, none of them had FEV_1_/FVC ratio less than 70%. From these, SAD was closely related to airway obstruction. Airway hyperresponsiveness was significantly associated with increased SAD (*p* = 0.021). As expected, MMEF had positive correlations with FEV_3_/FVC ratios (*r* = 0.68, *p* < 0.001), FEV_1_/FVC ratios (*r* = 0.78, *p* < 0.001) and percentage predicted PEF (*r* = 0.68, *p* < 0.001) (Additional file 1: Fig. S1). In addition, oxygen partial pressure was significantly lower in patients with SAD compared to patients without SAD.

### Risk factors for small airway dysfunction in pneumoconiosis

The stage III of pneumoconiosis had progressive massive fibrosis in accordance with the national criterion of the diagnosis of occupational pneumoconiosis [19, 22]. The stage III of asbestosis had more severe pulmonary fibrosis than stage I and II. Stage I and II were combined in the Logistic model in Table 4. In univariate Logistic regression analysis, aged 40 years and older, female sex, heavy smoking, BMI ≥ 25.0 kg/m^2^ and pneumoconiosis stage III were significantly associated with an increased risk of SAD in all patients (Table 4). In multivariable-adjusted analyses, the risk was significantly associated with these factors. In a subgroup analysis, cumulative pack-years, heavy smoking and current smokers were significantly associated with increased risk of SAD among males (Additional file 1: Fig. S2), but not among females. However, the number of smoking women was not enough to perform interaction analysis between sex and cigarette smoking.

**Table 4:** Logistic regression analysis for risk factors of small airway obstruction in the total patients with pneumoconiosis*

	Unadjusted			Adjusted		
	OR	95%CI	*p* Value	OR	95%CI	*p* Value
*Age, yrs*						
20–39	1.00	(ref)		1.00	(ref)	
40–59	2.34	1.22–4.49	0.010	2.02	1.02–3.98	0.043
≥ 60	3.35	1.74–6.45	< 0.001	3.20	1.55–6.62	0.002
*Sex*						
Men	1.00	(ref)		1.00	(ref)	
Women	1.57	1.16–2.11	0.003	1.76	1.17–2.64	0.007
*Smoking exposure, pack-yrs*						
0	1.00	(ref)		1.00	(ref)	
1–19	0.80	0.61–1.06	0.153	1.15	0.81–1.61	0.435
≥ 20	1.45	1.06–1.98	0.020	1.72	1.16–2.56	0.007
*BMI*^*‡*^*, kg/m*^*2*^						
< 18.5	1.74	0.55–5.47	0.343	1.58	0.49–5.01	0.448
18.5–24.9	1.00	(ref)		1.00	(ref)	
≥ 25.0	1.29	1.01–1.66	0.044	1.32	1.01–1.72	0.039
*Exposure duration, yrs*						
~ 4	1.00	(ref)		1.00	(ref)	
5–10	0.98	0.66–1.45	0.910	0.91	0.60–1.37	0.642
11–15	1.07	0.68–1.69	0.784	1.03	0.63–1.66	0.916
16 ~	0.95	0.65–1.39	0.796	0.74	0.50–1.11	0.148
*Types of pneumoconiosis*						
Asbestosis	1.00	(ref)		1.00	(ref)	
Silicosis	1.08	0.78–1.50	0.641	1.30	0.88–1.93	0.194
Coal workers’ pneumoconiosis	0.77	0.55–1.06	0.107	1.06	0.68–1.64	0.812
Other pneumoconiosis	0.69	0.45–1.04	0.078	1.23	0.75–2.09	0.381
*Stage of pneumoconiosis*						
I/II	1.00	(ref)		1.00	(ref)	
III	2.30	1.63–3.26	< 0.001	2.67	1.83–3.88	< 0.001

*OR* odds rate, *BMI* body-mass index

*All variables in the table were included in the multivariable model, while adjusting for exposure duration and types of pneumoconiosis

^‡^BMI was categorized as: underweight (< 18.5 kg/m^2^), normal (18.5–24.9 kg/m^2^) and overweight/obese (≥ 25.0 kg/m^2^)

In never-smokers, multivariable analysis showed that female sex (OR 1.78, 95% CI 1.10–2.88, *p* = 0.020), BMI ≥ 25.0 kg/m^2^ (OR 1.54, 95% CI 1.04–2.30, *p* = 0.032), pneumoconiosis stage II or stage III (OR 1.75, 95% CI 1.07–2.85, *p* = 0.026; OR 2.38, 95% CI 1.27–4.48, *p* = 0.007) were independent risk factors for development SAD (Additional file 1: Table S5).

## Discussion

In the present study, spirometric evaluation revealed SAD in the majority of pneumoconiosis regardless of its subtypes. Moreover, SAD was present across patients with all severities of pneumoconiosis. Of note, all patients with both pneumoconiosis and COPD were present SAD. Patients with SAD were older than patients without SAD and more likely to be women and heavy smokers. Importantly, patients with SAD had more airflow obstruction, air trapping, and diffusion dysfunction. Overall, aged 40 years and older, female sex, heavy smoking, BMI ≥ 25.0 kg/m^2^ and pneumoconiosis stage III were identified as independent risk factors for SAD in pneumoconiosis. Among never-smokers, those risk factors were not substantially altered.

Previous studies showed that the prevalence of SAD varied greatly, which used a very different definition of the conditions and focused primarily on the general population or individuals with established chronic respiratory diseases. A large cross-sectional study analysed data from 13,302 adults from the general population and showed that the prevalence of SAD was 6.3% in the United States [23]. The most recent national survey of SAD in China among 50,479 adults reported an overall prevalence of 43.5%, which was lower than that of pneumoconiosis (66.3%) in our cohort, and these two studies applied the same diagnostic criteria [9]. These findings suggested that a pervasive occurrence of SAD in pneumoconiosis might be a characteristic caused by occupational dust exposure. It was estimated that up to 74% of COPD patients had SAD (defined as R_5_–R_20_ > 0.07 kPa· s· L^−1^), while all patients with both pneumoconiosis and COPD were present SAD in our study [24]. Although these two studies were not directly comparable, the physiological changes associated with dust-related small airways might play a role in the development of COPD in pneumoconiosis. Even among pneumoconiosis with FEV_1_ ≥ 80% and FEV_1_/FVC ratio ≥ 70%, more than 40% of patients had SAD, supporting the theory that the effect of exposure to dusts on small airways is a primary response and independent from effects on the large airways [12].

Interestingly, our study showed that the prevalence of SAD was higher in females (73.4%) than in males (63.8%), consistent with findings from previous studies among a nationally representative population in China (42.1% in males and 57.9% in females) [9]. In our study, there were no significant sexes differences in smoking levels. Females had significantly shorter occupational exposure durations than males (median: 10.0 years vs. 13.0 years, *p* = 0.009). Thus, sex difference in the prevalence of SAD could be related to other factors apart from smoking levels or tenures. The explanation may be that females have higher levels of exposure to biomass fuels than males [25]. Biomass use has been also linked to an increased risk of SAD [9]. However, the reasons for the sex difference remain to be elucidated in future studies.

Although the overlap with smoking makes it difficult to assess the fraction of SAD attributable to occupational dust exposures, we found a similar prevalence of SAD among never-smokers and smokers, and it is likely that occupational dust exposure contributes significantly to the burden of SAD. Our study showed that the prevalence of SAD did not significantly differ among the various pneumoconiosis subtypes, indicating that SAD is a nonspecific reaction of small airways to mineral dust damage. Histologic observations showed that small airway lesions were very similar in workers exposed to a variety of different dusts, such as silica, asbestos, coal, iron oxide, and aluminium oxide [26, 27].

Several risk factors associated with SAD were identified in the present study. Cigarette smoking induced small airway inflammatory response, fibrosis and pigment deposition, which were associated with the decline in FEF 25–75%, FEV_1_/FVC and FEV_1_ [28]. Evidence indicated that occupational dust exposure induced structural changes in the small airway more than smoking alone [29, 30]. Smoking was linked to an increased risk of SAD in the present study, which was consistent with a study among the general population [9]. Nevertheless, smokers with SAD, who successfully quit smoking, seem to constantly improve their airway dysfunction as shown in a longitudinal study carried out in Belgium [31].Specifically, comprehensive strategies for smoking prevention and control should be implemented to reduce hazards. Being aged 40 years and older increased the risk of SAD, like previous findings reported in the general population [9]. The evaluation of normal airway morphology indicated that the thickness of small airway cartilage progressively decreased with older age, and the inner area correlated negatively with age [32]. Lung function decline was shown to be related to an increase in BMI, and the risk of SAD was significantly associated with an increase in BMI by 5 kg/m^2^ [9, 33].

Occupational dust exposure is known to cause pneumoconiosis and airway obstruction in both large and small airways [1, 2]. Exposure to a wide variety of mineral dusts leads to the development of COPD [2, 34]. SAD is a common early feature of COPD and a mechanism for COPD progression [35–37]. Persistent occupational dust exposure causes small airway disease as respirable particles travel to small airways and alveoli, where they are phagocytosed by macrophages, and increases the recruitment and activity of macrophages. This process upregulates several proinflammatory and profibrotic pathways, inducing inflammation and the subsequent repair/regeneration process, leading to tissue remodelling and eventually small airway loss [38, 39]. Small airway abnormalities were found preceding asbestosis or silicosis in animal models [40, 41]. Workers with lesions of pigmentation and fibrosis in the respiratory bronchioles had significantly reduced FEF 25–75%, FVC and FEV_1_ [29]. Abnormal small airways may be functionally significant even in the absence of pneumoconiosis [29]. These findings suggested that occupational dust exposure may contribute to SAD, which precedes demonstrable involvement of lung tissue.

To date, there is no gold standard specifically to assess SAD. The three measures (MMEF, FEF 50%, and FEF 75%) are relatively sensitive and objective to reflect SAD and are suitable for large-scale epidemiological studies [9]. In addition, our analyses revealed a significant correlation between MMEF and FEV_1_/FVC, consistent with the findings from other previous study [42]. The FEV_3_/FVC ratio is an often a neglected tool for identifying airflow limitations. Our study also found that the FEV_3_/FVC ratio was positively associated with MMEF. Clinically, the possible effects of mixed restrictive and obstructive lung function abnormalities on the presence of SAD remain to be determined.

Several limitations of the study need to be mentioned. First, a regional center for occupational medicine and worker's compensation is involved in Beijing Chaoyang Hospital, which represents a reference center for diagnosis and management of pneumoconiosis at city level. The patients with pneumoconiosis who have ever worked or lived in the city or transfer from other regions will see the doctors in the hospital. However, all patients from a single-medical center were enrolled, which may lead to bias to a certain extent. The analysis was based on a large number of pneumoconiosis patients with spirometry data, and standard spirometry was performed by the same technologist with the same instruments for the entire study population, which allowed a high internal comparability and reproducibility of our results. Second, SAD was defined according to spirometry, with the potential risk of underestimation in patients with traction bronchiectasis, especially patients with asbestosis. In the subgroup analysis, the prevalence of SAD in asbestosis was lower than that in other conditions, but there were no significant group differences. These results should be applied only to spirometry-defined SAD. Third, because of the lack of information on individual workplace environments or concentrations of dust, this study may lack the power to estimate the exposure–response relationship between cumulative exposure to dust and the prevalence of SAD. Fourth, only 14 females were ever-smokers, which was not enough to study the interactions between sex and cigarette smoking in dust exposure-induced pneumoconiosis. Finally, the study lacked longitudinal observations so associations cannot be attributed to being causal. The role of small airways in early disease and the prognosis of pneumoconiosis are still unknown and remain to be elucidated in future studies.

## Conclusions

In conclusion, the present study revealed a high prevalence of spirometry-defined SAD in Chinese patients with pneumoconiosis, regardless of its subtypes. Notably, all patients with both pneumoconiosis and COPD were present SAD. Aged 40 years and older, female sex, heavy smoking, BMI ≥ 25.0 kg/m^2^ and severe pneumoconiosis were the major risk factors for SAD. SAD may be one of the common functional abnormalities in early lung damage caused by occupational dust exposure. In addition to prevention of occupational exposure and smoking cessation, early detection of the presence of SAD may help to guide precautions and management of the disease.

## Supplementary Information

**Additional file 1.** Stages of pneumoconiosis based on chest radiograph.

## Data Availability

All data generated or analysed during this study are included in this published article and its supplementary information files.

## Abbreviations

BMI: Body-mass index
COPD: Chronic obstructive pulmonary disease
DLCO: Diffusing capacity of the lung for carbon monoxide
FEV_1_: Forced expiratory volume in 1 s
FEF 25% (50–75%): Forced expiratory flow at 25% (50–75%) of forced vital capacity
FVC: Forced vital capacity
MMEF: Maximal mid-expiratory flow
PEF: Peak expiratory flow
RV: Residual volume
SAD: Small airway dysfunction
TLC: Total lung capacity

## References

[1] Leung CC, Yu IT, Chen W (2012). Silicosis. Lancet.

[2] Perlman DM, Maier LA (2019). Occupational lung disease. Med Clin N Am.

[3] Hoy RF, Brims F (2017). Occupational lung diseases in Australia. Med J Aust.

[4] Nelson G, Girdler-Brown B, Ndlovu N, Murray J (2010). Three decades of silicosis: disease trends at autopsy in South African gold miners. Environ Health Perspect.

[5] Wu N, Xue C, Yu S, Ye Q (2020). Artificial stone-associated silicosis in China: a prospective comparison with natural stone-associated silicosis. Respirology.

[6] National Institute for Occupational Health (NIOH). http://news.zybw.com/xw/rdxw/15365.html.

[7] Global and regional burden of chronic respiratory disease in 2016 arising from non-infectious airborne occupational exposures: a systematic analysis for the Global Burden of Disease Study 2016. Occup Environ Med. 2020;77:142–50. 10.1136/oemed-2019-106013 PMC7035690 32054818

[8] Burgel PR (2011). The role of small airways in obstructive airway diseases. Eur Respir Rev.

[9] Xiao D, Chen Z, Wu S (2020). Prevalence and risk factors of small airway dysfunction, and association with smoking, in China: findings from a national cross-sectional study. Lancet Respir Med.

[10] Eurlings IM, Dentener MA, Cleutjens JP, Peutz CJ, Rohde GG, Wouters EF, Reynaert NL (2014). Similar matrix alterations in alveolar and small airway walls of COPD patients. Bmc Pulm Med.

[11] Hogg JC, Chu F, Utokaparch S (2004). The nature of small-airway obstruction in chronic obstructive pulmonary disease. N Engl J Med.

[12] de Jong K, Boezen HM, Kromhout H, Vermeulen R, Vonk JM, Postma DS (2014). Occupational exposure to vapors, gases, dusts, and fumes is associated with small airways obstruction. Am J Respir Crit Care Med.

[13] Long J, Stansbury RC, Petsonk EL (2015). Small airways involvement in coal mine dust lung disease. Semin Respir Crit Care Med.

[14] Fan Y, Xu W, Wang Y, Wang Y, Yu S, Ye Q (2020). Association of occupational dust exposure with combined chronic obstructive pulmonary disease and pneumoconiosis: a cross-sectional study in China. BMJ Open.

[15] Stansbury RC, Beeckman-Wagner LA, Wang ML, Hogg JP, Petsonk EL (2013). Rapid decline in lung function in coal miners: evidence of disease in small airways. Am J Ind Med.

[16] Seaton A, Lapp NL, Morgan WK (1972). Lung mechanics and frequency dependence of compliance in coal miners. J Clin Invest.

[17] Yang X, Yan Y, Xue C, Du X, Ye Q (2018). Association between increased small airway obstruction and asbestos exposure in patients with asbestosis. Clin Respir J.

[18] von Elm E, Altman DG, Egger M, Pocock SJ, Gotzsche PC, Vandenbroucke JP (2014). The Strengthening the Reporting of Observational Studies in Epidemiology (STROBE) statement: guidelines for reporting observational studies. Int J Surg.

[19] ILO (International Labour Office). International Classification of Radiographs of Pneumoconiosis. 2011. http://www.ilo.org/wcmsp5/groups/public/—ed_protect/—protrav/—safework/documents/publication/wcms_168260.pdf.

[20] Miller MR, Hankinson J, Brusasco V (2005). Standardisation of spirometry. Eur Respir J.

[21] Singh D, Agusti A, Anzueto A, et al. Global strategy for the diagnosis, management, and prevention of chronic obstructive lung disease: the GOLD science committee report 2019. Eur Respir J. 2019; 53. 10.1183/13993003.00164-2019 30846476

[22] National occupational health standard. Diagnosis of occupational pneumoconiosis (GBZ 70-2015). http://www.nhc.gov.cn/wjw/pyl/wsbz.shtml.

[23] Morris ZQ, Coz A, Starosta D (2013). An isolated reduction of the FEV3/FVC ratio is an indicator of mild lung injury. Chest.

[24] Crisafulli E, Pisi R, Aiello M, Vigna M, Tzani P, Torres A, Bertorelli G, Chetta A (2017). Prevalence of small-airway dysfunction among COPD patients with different GOLD stages and its role in the impact of disease. Respiration.

[25] Jindal S, Jindal A (2021). COPD in Biomass exposed nonsmokers: a different phenotype. Expert Rev Respir Med.

[26] Churg A, Wright JL (1983). Small-airway lesions in patients exposed to nonasbestos mineral dusts. Hum Pathol.

[27] Wright JL, Churg A (1984). Morphology of small-airway lesions in patients with asbestos exposure. Hum Pathol.

[28] Wright JL, Lawson LM, Pare PD, Wiggs BJ, Kennedy S, Hogg JC (1983). Morphology of peripheral airways in current smokers and ex-smokers. Am Rev Respir Dis.

[29] Churg A, Wright JL, Wiggs B, Pare PD, Lazar N (1985). Small airways disease and mineral dust exposure. Prevalence, structure, and function. Am Rev Respir Dis.

[30] Kennedy SM, Wright JL, Mullen JB, Pare PD, Hogg JC (1985). Pulmonary function and peripheral airway disease in patients with mineral dust or fume exposure. Am Rev Respir Dis.

[31] Verbanck S, Schuermans D, Paiva M, Meysman M, Vincken W (2006). Small airway function improvement after smoking cessation in smokers without airway obstruction. Am J Respir Crit Care Med.

[32] Su ZQ, Guan WJ, Li SY, Feng JX, Zhou ZQ, Chen Y, Zhong ML, Zhong NS (2019). Evaluation of the normal airway morphology using optical coherence tomography. Chest.

[33] Jones RL, Nzekwu MM (2006). The effects of body mass index on lung volumes. Chest.

[34] Hnizdo E, Vallyathan V (2003). Chronic obstructive pulmonary disease due to occupational exposure to silica dust: a review of epidemiological and pathological evidence. Occup Environ Med.

[35] Koo HK, Vasilescu DM, Booth S (2018). Small airways disease in mild and moderate chronic obstructive pulmonary disease: a cross-sectional study. Lancet Respir Med.

[36] Hogg JC, Pare PD, Hackett TL (2017). The contribution of small airway obstruction to the pathogenesis of chronic obstructive pulmonary disease. Physiol Rev.

[37] Abe K, Sugiura H, Hashimoto Y (2016). Possible role of Kruppel-like factor 5 in the remodeling of small airways and pulmonary vessels in chronic obstructive pulmonary disease. Respir Res.

[38] Mossman BT, Churg A (1998). Mechanisms in the pathogenesis of asbestosis and silicosis. Am J Respir Crit Care Med.

[39] Lopes-Pacheco M, Bandeira E, Morales MM (2016). Cell-based therapy for silicosis. Stem Cells Int.

[40] Begin R, Masse S, Bureau MA (1982). Morphologic features and function of the airways in early asbestosis in the sheep model. Am Rev Respir Dis.

[41] Churg A, Hobson J, Wright J (1989). Functional and morphologic comparison of silica- and elastase-induced airflow obstruction. Exp Lung Res.

[42] Stockley JA, Ismail AM, Hughes SM, Edgar R, Stockley RA, Sapey E (2017). Maximal mid-expiratory flow detects early lung disease in alpha1-antitrypsin deficiency. Eur Respir J.

