# Supplementary material for: Small airway dysfunction in pneumoconiosis: a cross-sectional study
Source: BMC Pulm Med. 2022 Apr 28;22:167. doi: 10.1186/s12890-022-01929-9 (PMC9052448; doi:10.1186/s12890-022-01929-9)
Supplement: Supplementary file 1 — Additional file 1. Stages of pneumoconiosis based on chest radiograph. [file 12890_2022_1929_MOESM1_ESM.pdf]

Small airway dysfunction in pneumoconiosis: a cross-sectional study

Yali Fan[1], Ruimin Ma[1], Xuqin Du[1], Dandan Chai[1], Shuangli Yang[1], QiaoYe[1]

[1]Clinical Center for Interstitial Lung Diseases, Beijing Institute of Respiratory Medicine, Department of Occupational Medicine and Toxicology, Beijing Chao-Yang Hospital, Capital Medical University, Beijing 100020, China

Methods

Stages of pneumoconiosis based on chest radiograph

Stage of pneumoconiosis was classified as follows: Stage I was shown by the highest density of small opacities was ≥1/0, the distribution affected two or more zones, and pleural plaques were apparent; Stage II was shown by the highest density of small opacities being ≥2/1, and the distribution affected more than four zones, or the highest density of small opacities was ≥3/2, and the distribution affected four or more zones; Stage III was shown by the highest density of small opacities being ≥3/2, and the distribution affected four or more zones with aggregation of small or large opacities, or the diameter of the largest opacity was ≥20 × 10 mm.

Table S1 Demographic characteristics of the 495 never-smokers and prevalence of small airway dysfunction in never-smokers

	Never-smokers		Prevalence of SAD		
	n	%	n	%	*p*-value
Overall	495	100	330	66.7	
Pneumoconiosis					0.147
Asbestosis	221	44.6	147	66.5	
Silicosis	150	30.3	108	72.0	
Coal workers' pneumoconiosis	84	17.0	48	57.1	
Other pneumoconiosis	40	8.1	27	67.5	
Age, yrs					**0.012**
20-39*	10	2.0	4	40.0	
40-49	90	18.2	50	55.6	
50-59	127	25.7	94	74.0	
60-69	151	30.5	98	64.9	
≥70	117	23.6	84	71.8	
Sex					**0.001**
Men	223	45.1	132	59.2	
Women	272	54.9	198	72.8	
BMI, kg/m^2					0.104
<18.5	5	1.0	4	80.0	
18.5-24.9	199	40.2	122	61.3	
≥25.0	291	58.8	204	70.1	
Duration of exposure, yrs					0.307
~4	74	14.9	45	60.8	
5-10	182	36.8	129	70.9	
11-15	72	14.5	50	69.4	
16~	167	33.7	106	63.5	
Stage of pneumoconiosis					**0.008**
I	298	60.2	183	61.4	
II	118	23.8	86	72.9	
III	79	16.0	61	77.2	

BMI, body-mass index, SAD, small airway dysfunction.

*Age groups 20-29 years and 30-39 years were combined because of small numbers of patients.

Table S2 Demographic characteristics of 1115 pneumoconiosis patients and prevalence of SAD with pre-bronchodilator values with $FEV_1 \geq 80\%$ and FEV_1/FVC ratio≥70%

	All		Prevalence of SAD		
	n	%	n	%	*p*-value
Overall	1115	100	237	21.3	
Pneumoconiosis					**0.015**
Asbestosis	339	30.4	68	20.4	
Silicosis	341	30.6	78	22.6	
Coal workers' pneumoconiosis	303	27.2	51	16.8	
Other pneumoconiosis	132	11.8	40	30.3	
Age, yrs					0.107
20-39	40	3.6	10	25.0	
40-49	223	20.0	59	26.5	
50-59	334	30.0	66	19.8	
60-69	315	28.3	59	18.7	
≥70	203	18.2	43	21.2	
Sex					**0.007**
Men	829	74.3	160	19.3	
Women	286	25.7	77	26.9	
Smoking history					0.178
Never-smoker	495	44.4	111	22.4	
Former smoker	312	28.0	55	23.1	
Current smoker	308	27.6	71	17.6	
Smoking exposure, pack-yrs					0.551
0	495	44.4	111	22.4	
1-19	372	33.4	79	21.2	
≥20	248	22.2	47	19.0	
BMI, kg/m^2					0.077
<18.5	16	1.4	0	0	
18.5-24.9	531	47.6	109	20.5	
≥25.0	568	50.9	128	22.5	
Duration of exposure, yrs					0.109
<5	168	15.1	32	19.0	
5-10	331	29.7	69	20.8	
11-15	166	14.9	47	28.3	
16~	450	40.4	89	19.8	
Stage of pneumoconiosis					**<0.001**
I	599	53.7	152	25.4	
II	286	25.7	63	22.0	
III	230	20.6	22	9.6	

SAD, small airway dysfunction; FVC, forced vital capacity; FEV_1, forced expired volume in the

first second; BMI, body-mass index.

*Age groups 20-29 years and 30-39 years were combined because of small numbers of patients.

Table S3 The prevalence of COPD, GOLD stage and small airway dysfunction in various subtypes of pneumoconiosis

	All	Asbestosis	Silicosis	Coal workers' pneumoconiosis	Other pneumoconiosis	*p*-value
n	1115	339	341	303	132	
COPD	345 (30.9)	63 (18.6)	131 (38.4)	119 (39.3)	32 (24.2)	<0.001
Classification of airflow limitation severity*						<0.001
GOLD stage I	94 (27.2)	6 (9.5)	44 (33.6)	35 (29.4)	9 (28.1)	
GOLD stage II	150 (43.5)	28 (44.4)	50 (38.2)	58 (48.7)	14 (43.8)	
GOLD stage III	83 (24.1)	26 (41.3)	32 (24.4)	18 (15.1)	7 (21.9)	
GOLD stage IV	18 (5.2)	3 (4.8)	5 (3.8)	8 (6.7)	2 (6.3)	
FEV_1 ≥80% predicted and FEV_1/FVC ≥70%	581 (52.1)	150 (44.2)	176 (51.6)	162 (53.5)	93 (70.5)	<0.001
SAD[§]	237 (40.8)	69 (46.0)	77 (43.8)	51 (31.5)	40 (43.0)	0.040
FVC ≥80% predicted and FEV_1/FVC ≥70%	598 (53.6)	157 (46.3)	178 (52.2)	171 (56.4)	92 (69.7)	<0.001
SAD[§]	268 (44.8)	84 (53.5)	83 (46.6)	60 (35.1)	41 (44.6)	0.009
FVC<80% predicted and FEV_1/FVC ≥70%	172 (15.4)	119 (35.1)	32 (9.4)	13 (4.3)	8 (6.1)	<0.001
SAD[§]	126 (73.3)	85 (71.4)	25 (78.1)	10 (76.9)	6 (75.0)	0.885
FVC ≥80% predicted and FEV_1/FVC <70%	215 (19.3)	18 (5.3)	94 (27.6)	86 (28.4)	17 (12.9)	<0.001
SAD[§]	215 (100.0)	18 (100.0)	94 (100.0)	86 (100.0)	17 (100.0)	
FVC <80% predicted and FEV_1/FVC <70%	130 (11.7)	45 (13.3)	37 (10.9)	33 (10.9)	15 (11.4)	0.739
SAD[§]	130 (100.0)	45 (100.0)	37 (100.0)	33 (100.0)	15 (100.0)	
AHR[‡]	579 (60.8)	71 (48.8)	157 (65.7)	290 (63.8)	61 (54.8)	0.252

Data was presented as n (%)

COPD, chronic obstructive pulmonary disease; GOLD, Global Initiative for Chronic Obstructive Lung Disease; FVC, forced vital capacity; FEV_1, forced expired volume in the first second; AHR, airway hyperresponsiveness; SAD, small airway dysfunction.

*GOLD stage I (mild COPD), FEV_1 ≥80% predicted; GOLD stage II (moderate COPD), FEV_1 ≥50% to <80% predicted; GOLD stage III (severe COPD), FEV_1 ≥30% to <50% predicted; GOLD stage IV (very severe), FEV_1 <30% predicted.

[§]The prevalence of small airway dysfunction in various pulmonary function states.

[‡]Bronchial challenge test was performed in patients with FEV_1 predicted more than 60%. Inpresent cohort of combined COPD and pneumoconiosis, 60.8% (579/952) patients were shown AHR.

Table S4 Comparison of pulmonary function tests between patients with or without small airway dysfunction in whole patients with pneumoconiosis

Variables	All	Small airway dysfunction		*p*-value
		Presence	Absence	
n	1115	739	376	
FVC, %pred	93.50 (77.80-106.80)	88.50 (72.60-102.90)	102.75 (89.63-112.58)	<0.001
FEV_1, %pred	86.10 (67.90-99.80)	76.10 (58.60-89.50)	101.65 (91.83-110.38)	<0.001
FEV_1/FVC, %	75.08 (67.42-80.64)	70.71 (62.83-75.68)	81.32 (78.17-84.61)	<0.001
FEV_3/FVC, %	92.54 (89.30-95.47)	90.71 (86.82-93.48)	95.21 (93.60-98.14)	<0.001
TLC, %pred	91.70 (79.80-102.20)	90.50 (78.28-102.43)	93.30 (81.75-100.85)	0.972
RV, %pred	101.20 (84.20-121.35)	105.80 (86.98-127.63)	93.60 (80.10-111.10)	<0.001
RV/TLC, %	41.38 (35.51-49.94)	44.80 (37.92-52.77)	36.56 (31.92-41.64)	<0.001
DLco SB, %pred	80.60 (62.65-95.98)	75.10 (58.70-90.85)	88.60 (74.20-102.70)	<0.001
PEF, %pred	91.10 (69.50-107.63)	80.40 (57.15-97.30)	107.75 (95.98-120.35)	<0.001
FEF25, %pred	78.60 (51.70-102.30)	63.50 (37.70-81.90)	107.40 (92.93-120.88)	<0.001
FEF50, %pred	57.60 (36.90-78.30)	45.00 (28.20-58.40)	86.50 (76.80-99.68)	<0.001
FEF75, %pred	45.10 (29.70-63.80)	35.30 (24.80-45.60)	71.75 (59.70-87.15)	<0.001
MMEF, %pred	55.20 (36.23-73.20)	42.05 (28.93-53.28)	79.85 (71.30-92.43)	<0.001
PaO_2*, mmHg	88.00 (82.00-95.00)	87.00 (80.00-93.00)	92.00 (86.00-98.00)	<0.001
CPI	17.99 (5.60-31.96)	20.92 (7.96-35.05)	13.56 (3.11-25.47)	<0.001
AHR‡ n (%)	579 (60.8)	382 (66.5)	197 (52.4)	0.021

Data was presented as n (%) or median (IQR).

SAD, small airway dysfunction; FVC, forced vital capacity; FEV_1, forced expired volume in the first second; TLC, total lung capacity; RV, residual volume; DLco SB, diffusion capacity for carbon monoxide of the lung single breath; PEF, peak expiratory flow; FEF25 (50-75), forced expiratory flow at 25% (50%-75%) of forced vital capacity; MMEF, maximal mid-expiratory flow; PaO_2, arterial partial pressure of oxygen; CPI, composite physiologic index; AHR, airway hyperresponsiveness; IQR, interquartile range.

*PaO_2 was in room air at rest.

‡Bronchial challenge test was performed in patients with FEV_1 predicted more than 60%. In present cohort of combined COPD and pneumoconiosis, 60.8% (579/952) patients were shown AHR.

Table S5 Logistic regression analysis for risk factors of small airway obstruction in 495 never-smokers*

	Unadjusted			Adjusted		
	OR	95%CI	*p*-value	OR	95%CI	*p*-value
Age, yrs						
20-39	1.00	(ref)		1.00	(ref)	
40-59	2.96	0.81-10.82	0.101	2.83	0.73-10.95	0.133
≥60	3.17	0.87-11.54	0.079	3.52	0.88-14.15	0.074
Sex						
Men	1.00	(ref)		1.00	(ref)	
Women	1.85	1.26-2.69	**0.001**	1.78	1.10-2.88	**0.020**
BMI‡, kg/m^2						
<18.5	2.53	0.28-23.01	0.411	1.68	0.18-15.71	0.650
18.5-24.9	1.00	(ref)		1.00	(ref)	
≥25.0	1.48	1.01-2.17	**0.043**	1.54	1.04-2.30	**0.032**
Exposure duration, yrs						
~4	1.00	(ref)		1.00	(ref)	
5-10	1.57	0.89-2.76	0.119	1.41	0.78-2.57	0.258
11-15	1.47	0.74-2.91	0.275	1.19	0.56-2.50	0.652
16~	1.12	0.64-1.97	0.694	0.94	0.51-1.70	0.826
Types of pneumoconiosis						
Asbestosis	1.00	(ref)		1.00	(ref)	
Silicosis	1.29	0.82-2.04	0.264	1.48	0.82-2.68	0.190
Coal workers' pneumoconiosis	0.67	0.40-1.12	0.129	1.12	0.54-2.30	0.768
Other pneumoconiosis	1.05	0.51-2.14	0.903	1.95	0.82-4.61	0.129
Stage of pneumoconiosis						
I	1.00	(ref)		1.00	(ref)	
II	1.69	1.06-2.70	**0.028**	1.75	1.07-2.85	**0.026**
III	2.13	1.20-3.79	**<0.010**	2.38	1.27-4.48	**0.007**

SAD, small airway dysfunction; OR, odds rate; BMI, body-mass index.

*All variables in the table were included in the multivariable model, while adjusting for age, exposure duration and types of pneumoconiosis.

‡The patients with BMI <18.5 kg/m^2 means underweight, 18.5-24.9 kg/m^2 means normal range, and ≥25.0 kg/m^2 means overweight and obese.

Figure S1 Correlations by the Spearman test between percentage predicted MMEF and other four pulmonary function parameters (A) FEV_3/FVC ratio, (B) FEV_1/FVC ratio, (C) percentage predicted PEF and (D) RV/TLC ratio in the total patients with pneumoconiosis

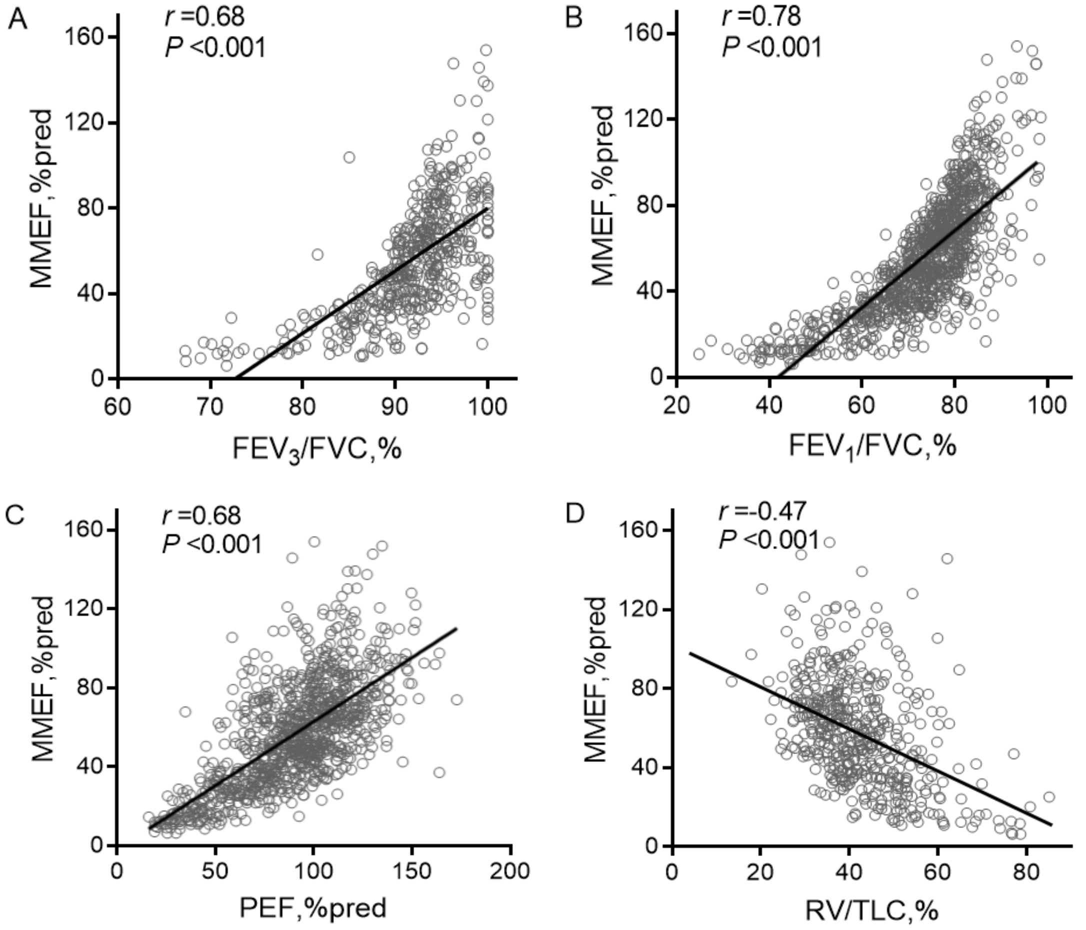

MMEF, maximal mid-expiratory flow; FEV_1/FVC, forced expired volume in 1s to forced vital capacity ratio; FEV_3/FVC, forced expiratory volume in 3s to forced vital capacity ratio; RV/TLC, residual volume to total lung capacity ratio; PEF, peak expiratory flow; %pred, percentage predicted.

Figure S2 Forest plot showing odds ratio for smoking exposure and small airway dysfunction in males

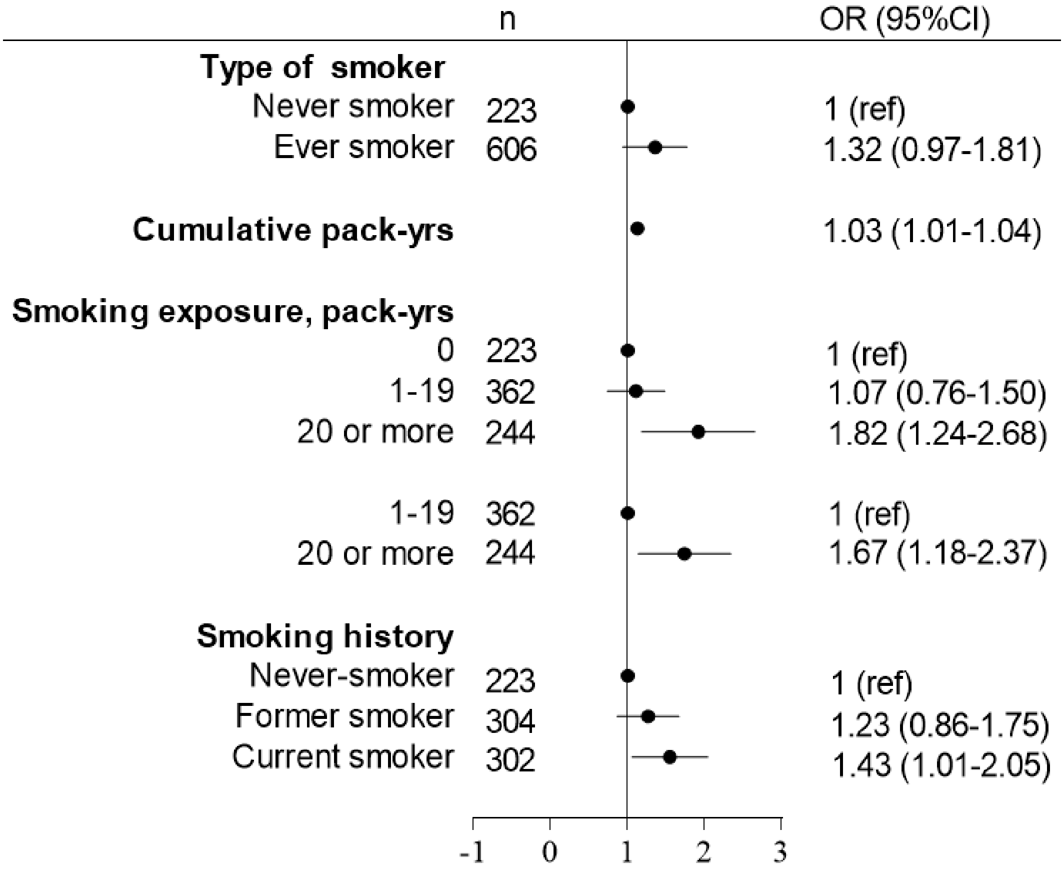

OR, odds ratio.

ORs were adjusted for age, smoking exposure, BMI, the duration of exposure, subtypes and stages of pneumoconiosis.
